# Sigma-1 Receptor Activation Induces Autophagy and Increases Proteostasis Capacity In Vitro and In Vivo

**DOI:** 10.3390/cells8030211

**Published:** 2019-03-02

**Authors:** Maximilian G. Christ, Heike Huesmann, Heike Nagel, Andreas Kern, Christian Behl

**Affiliations:** Institute of Pathobiochemistry, University Medical Center of the Johannes Gutenberg University, Duesbergweg 6, 55128 Mainz, Germany; maxchris@uni-mainz.de (M.G.C.); duerk@uni-mainz.de (H.H.); nagelh@uni-mainz.de (H.N.); akern@uni-mainz.de (A.K.)

**Keywords:** sigma-1 receptor, autophagy, proteostasis, neurodegeneration, *C. elegans*

## Abstract

Dysfunction of autophagy and disturbed protein homeostasis are linked to the pathogenesis of human neurodegenerative diseases and the modulation of autophagy as the protein clearance process has become one key pharmacological target. Due to the role of sigma-1 receptors (Sig-1R) in learning and memory, and the described pleiotropic neuroprotective effects in various experimental paradigms, Sig-1R activation is recognized as one potential approach for prevention and therapy of neurodegeneration and, interestingly, in amyotrophic lateral sclerosis associated with mutated Sig-1R, autophagy is disturbed. Here we analyzed the effects of tetrahydro-*N*,*N*-dimethyl-2,2-diphenyl-3-furanmethanamine hydrochloride (ANAVEX2-73), a muscarinic receptor ligand and Sig-1R agonist, on autophagy and proteostasis. We describe, at the molecular level, for the first time, that pharmacological Sig-1R activation a) enhances the autophagic flux in human cells and in *Caenorhabditis elegans* and b) increases proteostasis capacity, ultimately ameliorating paralysis caused by protein aggregation in *C. elegans*. ANAVEX2-73 is already in clinical investigation for the treatment of Alzheimer’s disease, and the novel activities of this compound on autophagy and proteostasis described here may have consequences for the use and further development of the Sig-1R as a drug target in the future. Moreover, our study defines the Sig-1R as an upstream modulator of canonical autophagy, which may have further implications for various conditions with dysfunctional autophagy, besides neurodegeneration.

## 1. Introduction

The pathogenesis of neurodegenerative disorders, including Alzheimer’s and Parkinson’s disease (AD, PD) as well as amyotrophic lateral sclerosis (ALS), has been linked to a disturbed protein homeostasis [1,2,3]. Therefore, the control and maintenance of proteome integrity and proteostasis is of utmost importance. Cellular proteostasis includes protein folding, protein assembly, refolding of damaged proteins, as well as protein degradation, and is under the control of a fine-tuned network of factors, including chaperones, such as heat shock protein 70 (HSP70), and distinct co-chaperones [4]. For intact function and long-term survival of the cell it is crucial to remove misfolded proteins via specialized processes; the two major cellular degradation pathways are the ubiquitin proteasome system (UPS) and autophagy [5,6,7]. The UPS is of particular importance for the physiological protein turnover, but is limited in the degradation substrates, and the autophagic-lysosomal pathway is responsible for the clearance of aggregated and disease-associated proteins, especially under pathogenic and aging conditions.

Autophagy is a highly dynamic vesicle-mediated cellular degradation pathway involving double-membraned vesicles, called autophagosomes, which sequester large protein complexes (protein aggregates), and even whole organelles, and deliver them to lysosomes for degradation [8]. Under low nutrition and energy conditions, autophagy guarantees energy supply by generating amino acid building blocks via recycling. In addition, autophagy plays an important role as a stress and adaptive response and rescue mechanism to maintain cell survival and function [8]. Canonical autophagy responds to environmental cues via a variety of factors that mainly belong to homologs of autophagy-related (ATG) genes, originally identified in yeast [9]. The mammalian target of rapamycin mTOR complex 1 (mTORC1) negatively regulates autophagic activity via inhibitory phosphorylation of ULK1, and is the key initial regulator of canonical autophagy. More downstream membrane expansion is modulated by two ubiquitin-like conjugation systems (ATG12-ATG5 and ATG8/LC3) and the ATG18 protein family members of WD repeat domain phosphoinositide interacting 1-4 (WIPI1-4), as recently excellently reviewed [10].

There is a great amount of data linking dysfunction and malfunction of autophagy to neurodegenerative disease and, consistent with its role in proteostasis, to the accumulation of protein aggregates. Thus, the modulation of autophagy has become one key pharmacological target in neurodegeneration [11,12,13]. In fact, there are multiple overlaps of autophagy and pathogenesis pathways in AD, PD, and ALS [12]. Recently different alternative views and new pharmacological targets towards AD prevention and treatment are evolving, and include a strong focus on the autophagy process.

There are two subtypes of sigma receptors, sigma receptor-1 and sigma receptor-2, both highly expressed in the central nervous system; sigma-1 receptor is present also in various tumor cell lines, including HEK293 and HeLa cells employed in this study [14,15,16]. Sigma-1 receptor (Sig-1R) was cloned in 1996 [17,18] and represents an integral membrane protein of 223 amino acids localized to the endoplasmic reticulum (ER) (and the ER–mitochondria interface) suggesting a role as ER chaperone. Sig-1R was shown to promote cellular survival by (1) ensuring Ca^2+^ signaling from the ER into mitochondria; (2) enhancing the signaling of ER to the nucleus; and (3) attenuating free radical damage by modulation of the activity of Nrf2, a redox-responsive transcription factor [19,20]. Structurally, Sig-1R ligand binding is characterized [21] and the crystal structure of the human receptor is solved [22].

In general, deficits in Sig-1R expression or activity are linked to neurodegeneration and the activation of Sig-1R is associated with neuroprotection in different in vitro and in vivo models, employing different types of pharmacological Sig-1R activators with different pharmacological profiles [15,23]. The pharmacological activation of Sig-1R leads to pluripotent modulatory downstream effects, and incorrect function of Sig-1R is strongly suggested to be also involved in the pathogenesis of neurodegeneration [24]. This is the basis of an effort to design novel and highly specific pharmacological Sig-1R activators for the therapy of neurodegenerative disease, including AD [25].

In this context a novel Sig-1R agonist, tetrahydro-*N*,*N*-dimethyl-2,2-diphenyl-3-furanmethanamine hydrochloride (ANAVEX2-73), was developed. Pharmacologically, ANAVEX2-73 shows a dual activity on Sig-1 as well as on muscarinic receptors, acting with described affinities in the low micromolar range [26,27]. Previously, pre-clinical studies in animal models demonstrated robust disease-modifying activities of ANAVEX2-73. Regarding AD, ANAVEX2-73 has undergone testing in Phase 2a trial of patients demonstrating a favorable safety profile and a concentration-dependent improvement against exploratory endpoints [28,29,30]. A variety of neuromodulatory and neuroprotective effects are also already known for ANAVEX2-73, including mitochondrial protection in mouse models of AD, regulation of ERK activation and promotion of survival of astrocytes, as well as protection against oxidative stress [31,32,33].

First evidence for a possible link of Sig-1R, autophagy, and neurodegeneration has been recently shown in the context of ALS. It was discovered that ALS-linked mutant Sig1-R causes an accumulation of autophagic material and actually reduced autophagy [34], and that mice with genetically-altered Sig-1R show defective autophagy [35]. Moreover, it was demonstrated that cocaine-mediated autophagy in astrocytes involves Sig-1R [36]. In addition, it was found that a small-molecule Sig-1R modulator induces autophagic degradation of programmed-death ligand 1 (PD-L1) in cancer cells [37]. These findings prompted us to study the potential of ANAVEX2-73 to have an effect on autophagy in human HeLa and HEK293 cells (in vitro) as well as in *C. elegans* (in vivo), employing standard measures to analyze autophagic activity [38,39,40,41]. Moreover, the effects of ANAVEX2-73 on protein aggregation and, subsequently, the impact of protein aggregates on movement behavior in *C. elegans* were studied. Excitingly, ANAVEX2-73 is a potent inducer of autophagic flux in vitro and in vivo and ameliorates protein aggregate formation and paralysis in *C. elegans*.

## 2. Materials and Methods

### 2.1. Cell Culture and Microscopy

HeLa and HEK293A cells were cultured in DMEM (Invitrogen, Carlsbad, CA, USA, 41965062) supplemented with active FBS (Life Technologies GmbH, Carlsbad, CA, USA, 10270106), 1× ABAM (Invitrogen, 15240-062) and 1 mM sodium pyruvate (Invitrogen, 1136-088). After medium change, they were treated for 2 h with 10, 1, and 0.1 µM ANAVEX2-73 and PRE-084 (Tocris, Bristol, UK, 0589), respectively; ANAVEX2-73 was provided by ANAVEX Life Sciences Corp, New York, NY, USA. Afterwards Bafilomycin A_1_ (BafiA_1_) (Toronto Research Chemicals, North York, ON, Canada, B110000) was added for a further 2 h and the cells were eventually harvested. Western blot analyses were performed as described previously [40,41]. Briefly, cells were subjected to SDS–PAGE using precast NuPAGE 4%–12% Bis-Tris gels (Invitrogen, NPO322). Proteins were detected by chemiluminescence using the Amersham Imager 600 (GE).

Confocal fluorescence microscopical analyses of HEK293A cells stably expressing GFP-LC3B (kind gift of Dr. Sharon Tooze) were performed with the laser scanning microscope LSM 710 (Zeiss, Oberkochen, Germany).

### 2.2. C. elegans Strains, Maintenance, and Methods

*C. elegans* were maintained according to standard procedures on nematode growth medium (NGM) plates seeded with HB101 *Escherichia coli*. The following strains were employed in this study: GFP-LGG-1 (ex[Plgg1-lgg1-GFP]/pRF4; kind gift of Beth Levine), maintained at 20 °C; and the strain CL2006 (dvIs2 [pCL12(unc-54/human Aβ peptide 1-42) + pRF4]), maintained at 15 °C, as previously described [40,42]. The latter strain was obtained from the Caenorhabditis Genetic Center (USA).

For analysis of paralysis rate, synchronous CL2006 nematodes were cultivated at 15 °C on plates seeded with HB101 *E. coli*, resuspended in M9 buffer (control) or 100 µM or 50 µM ANAVEX2-73, respectively. Starting at the first day of adulthood, worms were transferred to fresh plates daily and were tested for paralysis by tapping their nose with a platinum wire. Worms that moved their nose but failed to move their bodies were scored as paralyzed. Dead worms or worms showing other phenotypes were not included into the statistics. Staining of amyloid β42 aggregates using thioflavine S (Sigma T1892) were carried out as previously described [40]. Worms were mounted on 2% agar pads on a glass slide and confocal fluorescence microscopical analyses were performed with the laser scanning microscope LSM 710 (Zeiss, Oberkochen).

For analysis of autophagic activity, synchronous nematodes expressing GFP-LGG-1 were cultivated at 20 °C. At first day of adulthood, worms were transferred to 80 µM ANAVEX2-73 or control M9 liquid culture medium for 2 h, and were subsequently treated with BafiA_1_ or dimethyl sulfoxide (DMSO) (control) for 2 h. Thereafter, worms were lysed for Western blotting or analyzed by confocal fluorescence microscopy.

Western blot analyses were performed as described previously [40]. Generally, 12 worms were subjected to SDS–PAGE using precast NuPAGE 4%–12% Bis-Tris gels (Invitrogen, NPO322). Proteins were detected by chemiluminescence using the Amersham Imager 600 (GE).

### 2.3. Quantitative Real-Time PCR

RNA extraction, reverse transcription, and real-time PCR were performed as described previously [40].

### 2.4. Statistical Methods

Statistical significance was determined by Student t-test using SIGMA STAT (SPSS Science) as well as the log-rank test using SPSS Statistics (IBM). Statistical significance was accepted at a level of *p* ≤ 0.05. The results are expressed as mean ± standard deviation (SD).

## 3. Results and Discussion

### 3.1. Sig1-R Agonist ANAVEX2-73 Enhances Autophagic Activity

**Figure 1:**
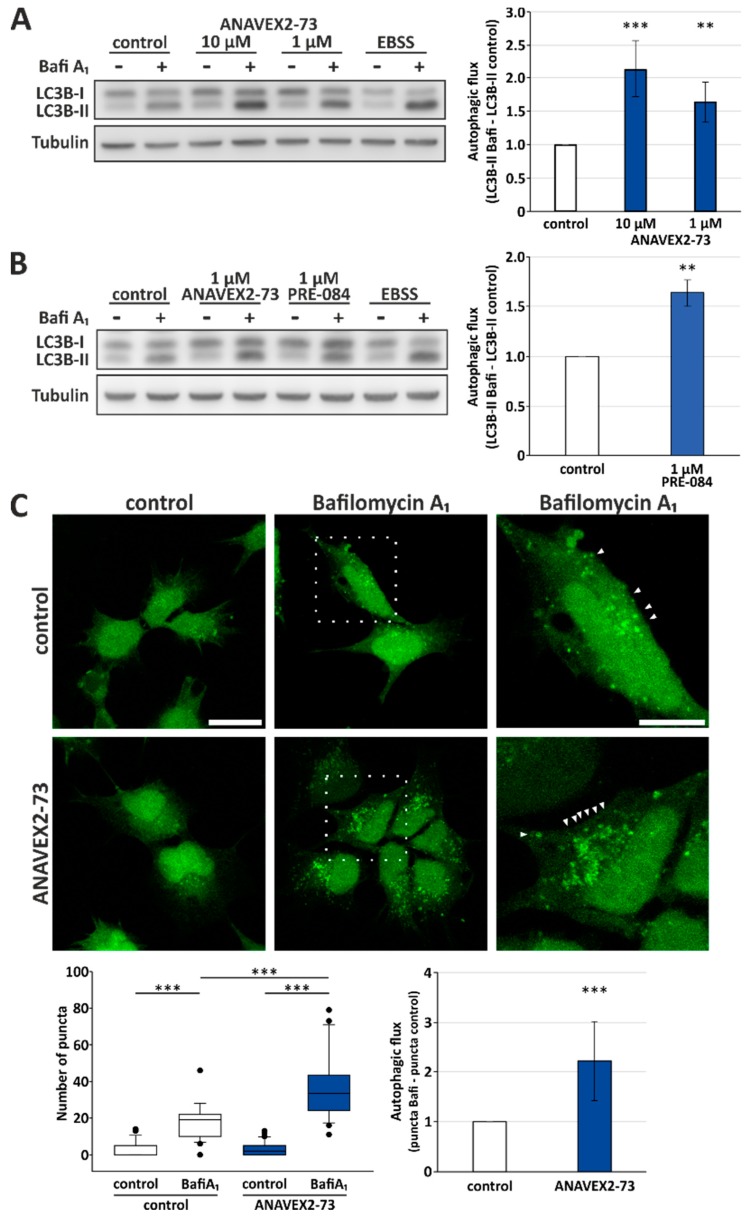
Sig-1R activation enhances autophagic activity. Western blot analyses of the autophagic flux upon addition of ANAVEX2-73 (**A**) or PRE-084 (**B**). HeLa cells were treated with indicated concentrations of ANAVEX2-73, PRE-084, and BafiA_1_ (2 μM) or DMSO. Statistics are depicted as mean +/− SD. *** *p* ≤ 0.001, ** *p* ≤ 0.01, *t*-test, *n* = 4. (**C**) Representative confocal fluorescence microscopic images of HEK293 cells stably transfected with a GFP-LC3B reporter construct. Cells were treated with 1 µM ANAVEX2-73, BafiA_1_, and/or DMSO. Scale bar = 20 µm or 10 µm, respectively. GFP-positive autophagosomal structures (indicated by arrowheads) were counted in approx. thirty cells per treatment in three independent experiments. *** *p* ≤ 0.001, *t*-test.

To study the effect of ANAVEX2-73 on autophagy, we treated human HeLa cells with the compound and analyzed autophagic activity by investigating the flux of LC3-II. LC3-II is the lipidated form of LC3, which (partially) stays attached to autophagosomes and thus gets degraded by lysosomes. Therefore, the quantification of the LC3-II flux, using BafiA_1_ for inhibition of lysosomal degradation, directly corresponds to cellular autophagic activity following the appropriate guidelines [38]. As displayed in Figure 1, ANAVEX2-73 significantly induces autophagic flux when compared to control conditions. There is a concentration-dependent and significant increase in the autophagic flux following application of ANAVEX2-73: An increase of over 2-fold at 10 µM and over 1.5-fold at 1 µM ANAVEX2-73 (Figure 1A). As standard positive control to provoke the induction of autophagy, HeLa cells were incubated with EBSS medium, which resembles nutrient deprivation as autophagy stimulus.

Here we focused on ANAVEX2-73 as Sig-1R agonist but, of course, other common and highly selective Sig-1R agonists are available and were already studied in different cellular and animal models. Such compounds include (+)-pentazocine, (+)-SKF10,047, SA4503 (1-[2-(3,4-dimethoxyphenyl)ethyl]-4-(3-phenylpropyl)piperazine), and PRE-084 (2-morpholin-4-ylethyl 1-phenylcyclohexane-1-carboxylate) [14]. Since the Sig-1R ligand PRE-084 shows various activities in the central nervous system in animal models, such as nootropic and antidepressant activities [43], we included this compound in some of the flux assays as control. Interestingly, we found that PRE-084 also promotes autophagic activity in HeLa cells; PRE-084 induces the autophagic flux comparable to ANAVEX2-73: at 1 μM an over 1,5-fold induction of the autophagic flux was observed (Figure 1B). But in contrast to ANAVEX2-73, PRE-084 and the other experimental compounds are not applicable in clinical studies.

Next, the Western blot experiments were complemented by direct visualization of the extent of autophagosome appearance in HEK293 cells. To do so, we applied ANAVEX2-73 to HEK293 cells stably expressing a GFP-LC3B reporter construct. This cell model allows direct monitoring of the accumulation of LC3-II-positive autophagosomal structures upon BafiA_1_ supplementation by confocal fluorescence microscopy [44]. Indeed, ANAVEX2-73 treatment resulted in an overall increased number of LC3-II-positive puncta and thus autophagic flux (Figure 1C).

Taken together, in both independent cell assays and in two different human cell lines, Sig-1R activation induced a significantly increased autophagic flux. Of course, it needs to be considered that part of the effect of ANAVEX2-73 as Sig-1R ligand could potentially be ascribed to its effects at the muscarinic ACh-receptor. But not much is known about the impact of mACh receptors on autophagy. In fact, so far there is only one report in the literature showing that ACh-induced autophagy has cytoprotective effects through the muscarinic ACh-receptor activated-AMPK-mTOR pathway [45]. On the other hand, our finding that also PRE-084, as an exclusive selective Sig-1R agonist, was inducing autophagic flux, strongly supports ANAVEX2-73′s effects on autophagy as being mediated by Sig-1R activation. Moreover, no experimental data exist that an activation of the muscarinic ACh-receptor has beneficial effects on protein aggregation and proteostasis, as clearly ANAVEX2-73 has, as shown below.

### 3.2. Sig-1R Activation Induces ULK1 Phosphorylation and Affects Expression Levels of Distinct Autophagy Network Factors

**Figure 2:**
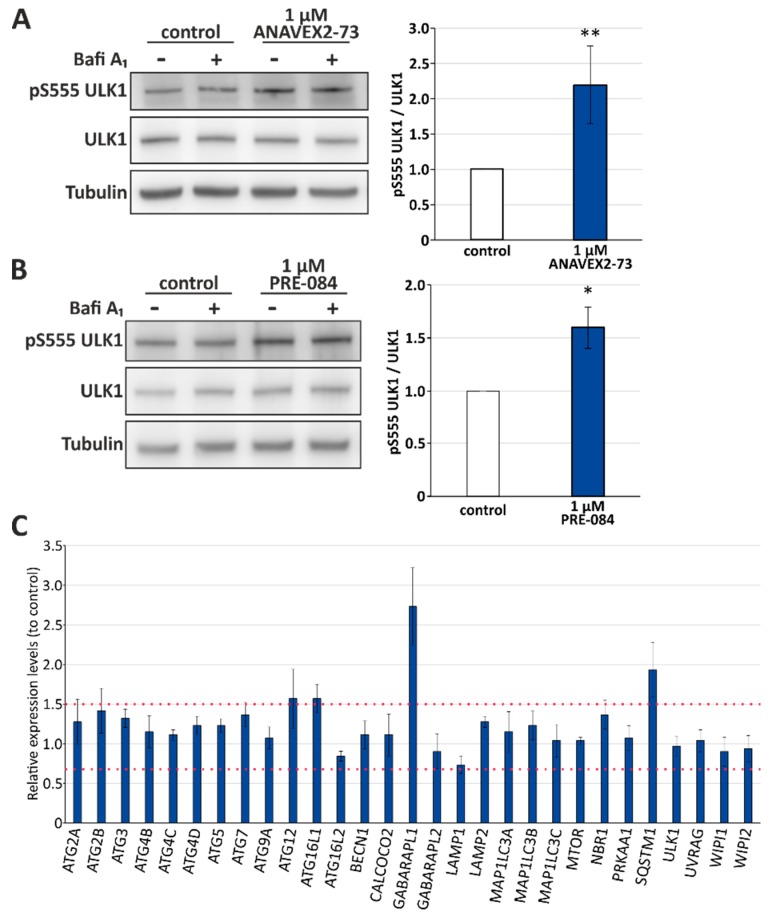
Sig-1R activation stimulates ULK1 activation and affects expression levels of distinct autophagy network factors. (**A**) Western blot analyses of ULK1 phosphorylation at serine 555 (pS555) upon treatment of HeLa cells with 1 µM ANAVEX2-73. Statistics are depicted as mean ± SD. ** *p* ≤ 0.01, *t*-test, *n* = 4. (**B**) Western blot analyses of ULK1 phosphorylation at serine 555 (pS555) upon treatment of HeLa cells with 1 µM PRE-084. Statistics are depicted as mean ± SD. * *p* ≤ 0.05, *t*-test, *n* = 4. (**C**) Relative expression levels of autophagy network factors were analyzed employing the autophagy qPCR array. The expression of each gene is depicted in relation to control cells (set to 1) and the threshold for up- or down-regulation is defined as 1.5 and 0.67, respectively.

Activation of the serine/threonine protein kinase ULK1 (unc-51-like kinase 1) via phosphorylation at serine 555 indicates stimulation of the canonical autophagy pathway. ANAVEX2-73 significantly induced ULK1 serine 555 phosphorylation (up to 2-fold at 1 µM; Figure 2A). Again, we analyzed also PRE-084 as Sig-1R agonist and found that it promotes ULK1 serine 555 phosphorylation to a similar extend (up to 1.5-fold at 1 µM; Figure 2B). It has to be mentioned that this activating ULK1 phosphorylation can be inhibited by mTOR as well as stimulated via AMPK kinase [46], both are basal physiological sensors of nutritional conditions and key signal transducers of canonical autophagy stimulation. ULK1 is actually the signal mediating the induction of the formation of the phagophore during the autophagy process and therefore, a central promoter of autophagy. ULK1 itself functions in a complex with at least three protein partners: FIP200 (focal adhesion kinase family interacting protein of 200 kDa), ATG13, and ATG101. Since a complex pattern of upstream pathways (including mTOR and AMPK) converge on ULK1, it suggests this complex acts as a node converting multiple signals into autophagosome formation [47].

Since we found that Sig-1R activation significantly induces ULK1 phosphorylation and autophagic flux, next, we investigated relative expression levels of key autophagy network factors, representing different setpoints in the autophagy process after treatment of HeLa cells with ANAVEX2-73, employing a qPCR autophagy array (Figure 2C). Most prominently, we found an ANAVEX2-73-mediated induction of the mRNA expression of GABA Type A Receptor Associated Protein Like 1 (GABARAPL1; approximately 2.7-fold increased expression; cut-off for induction was set at the expression level of 1.5), which, like GABARAP, associates with autophagic vesicles and is involved in the autophagy process [48].

GABARAPL1 belongs to the human MAP1LC3 family consisting of six ATG8 orthologs, MAP1LC3A, MAP1LC3B, MAP1LC3C, and three MAP1LC3 paralogs, the GABA receptor-associated proteins GABARAP1, GABARAPL1, and GABARAPL2, with partially redundant roles in autophagy [38]. In addition, the expression of the ubiquitin and autophagy receptor SQSTM1/p62 involved in selective macroautophagy pathways was enhanced by ANAVEX2-73 (expression level of approx. 1.9). Moreover, there was also a clear tendency towards the induction of ATG12, which is conjugated to ATG5 and is building an autophagosomal protein complex that finally acts together with ATG16L1 in autophagosomal biogenesis [49]; consistently, the expression of ATG16L1 appeared also slightly enhanced following treatment of the cells with ANAVEX2-73 (Figure 2C). Moreover, it is obvious that none of the autophagy network factors included in this qPCR array were downregulated in their expression upon treatment with ANAVEX2-73, supporting the key finding that Sig-1R activation has a positive modulatory effect on autophagy.

### 3.3. ANAVEX2-72 Positively Regulates Autophagy, Increases Proteostasis Capacity, and Improves Protein Aggregation-Mediated Paralysis in C. elegans

**Figure 3:**
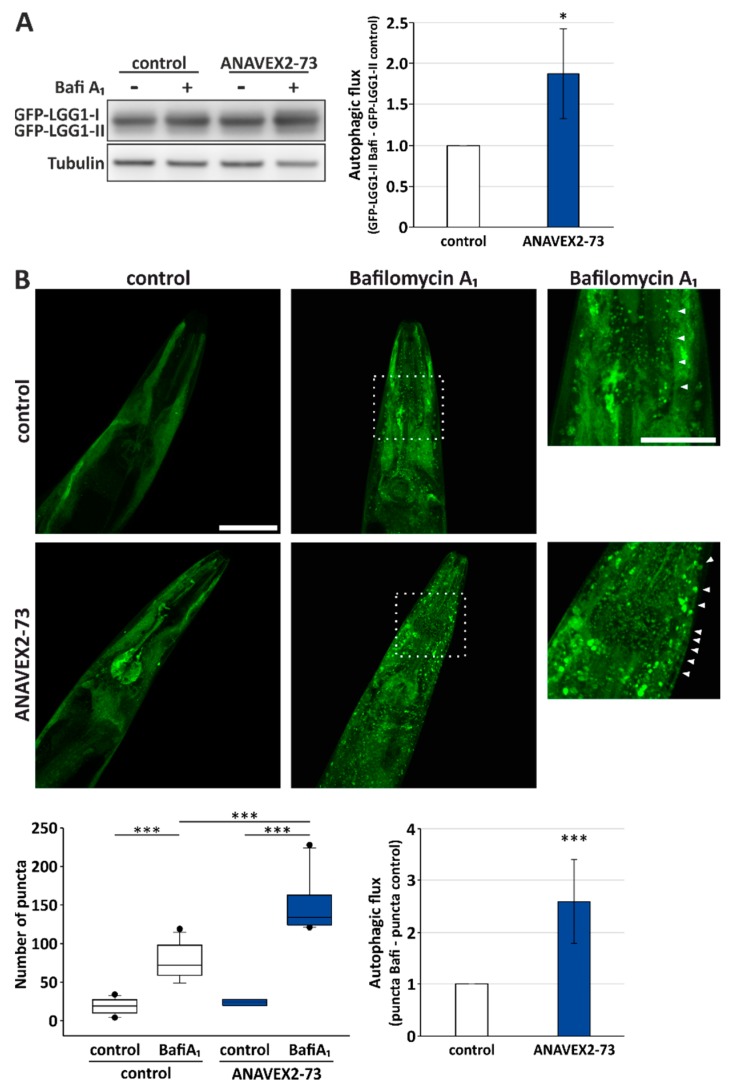
Sig-1R activation by ANAVEX2-73 enhances autophagy in *C. elegans*. (**A**) Western blot analyses of GFP-LGG1 after treatment of worms with 80 µM ANAVEX2-73. Nematodes were treated with BafiA_1_ or DMSO for 6 h. Statistics are depicted as mean ± SD. * *p* ≤ 0.05, *t*-test, *n* = 3. (**B**) Representative confocal fluorescence microscopic images of *C. elegans* treated with 80 µM ANAVEX2-73 and BafiA_1_ or DMSO (for a total of 2 h). Scale bar = 50 and 25 μm. The number of GFP-positive autophagosomal structures (indicated by arrowheads) were counted in three independent experiments and in each experiment in at least 8–11 respective head regions of worms. Autophagic flux was calculated as indicated, comparing GFP-positive puncta plus BafiA_1_ with puncta in the controls *** *p* ≤ 0.001, *t*-test.

Autophagy modulation by ANAVEX2-73 in vitro, and its impact on some key autophagy network factors, prompted us to further analyze the impact of Sig-1R activation by ANAVEX2-73 on autophagy and proteostasis in vivo, employing *C. elegans*. The nematode ortholog of the human Sig-1R is W08F4.3 and is expressed in several tissues, including the muscular system. To monitor autophagic flux in vivo, we employed a GFP-LGG-1 reporter worm strain. LGG-1 is a nematode ortholog of the mammalian GABARAP, and the GFP-tagged protein can be used to evaluate autophagic activity by Western blotting as well as confocal fluorescence microscopy. Employing Western blotting, we analyzed the levels of GFP-LGG-1-II plus BafiA_1_ and without BafiA_1_, analogously to the flux measurements in HeLa cells, as shown in Figure 1. Indeed, ANAVEX2-73 significantly enhanced autophagic flux in *C. elegans* almost 2-fold (Figure 3A). To further substantiate this finding we used confocal fluorescence microscopy to directly visualize autophagosomal structures, as indicated by GFP-LGG-1-positive puncta [40]. ANAVEX2-73 supplementation (plus/minus BafiA_1_) significantly increased the number of GFP-LGG1 puncta, which is indicative of increased autophagic activity; treatment of worms with ANAVEX2-73 lead to a relative increase in numbers of puncta after BafiA_1_ treatment when compared to control worms. In fact, we found a significant increase; autophagic flux as observed in vivo is induced by ANAVEX2-73 by approx. 2,5-fold (Figure 3B), which is consistent with the Western blot analysis (Figure 3A).

**Figure 4:**
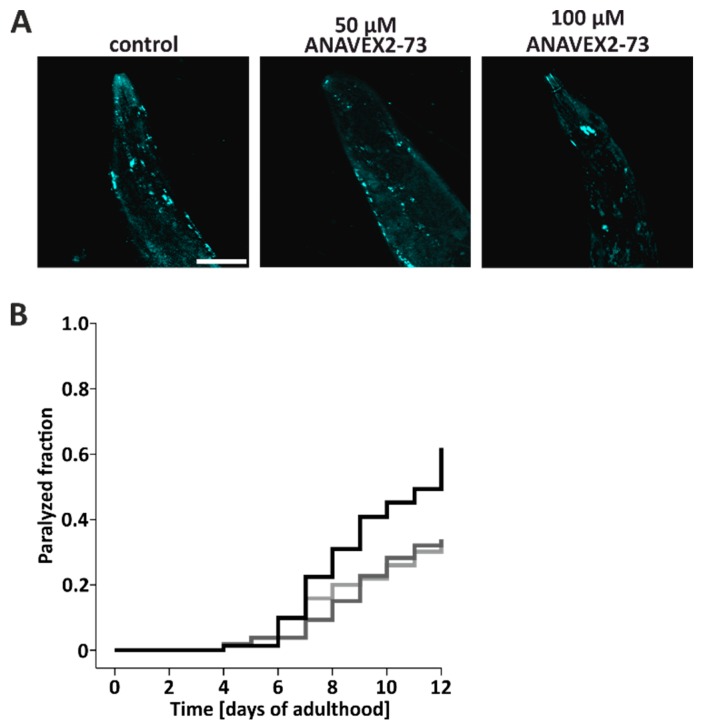
Sig-1R activation by ANAVEX2-73 increases proteostasis capacity in *C. elegans* and ameliorates Aβ42-caused paralysis. (**A**) Representative confocal fluorescence microscopic images of Thioflavin S-positive Aβ42 aggregates in head regions of nematodes, treated with 80 µM ANAVEX2-73 or M9 medium (control), for nine consecutive days. Scale bar = 50 µm. (**B**) Analyses of Aβ42-induced paralysis. Worms were maintained in the presence of ANAVEX2-73 or M9 buffer and the paralysis phenotype was examined daily. Statistics were conducted using the log-rank test. The paralyzed fraction is significantly different comparing ANAVEX2-73 treated and control worms. Three independent experiments with a total of approx. 70 worms per treatment. Black = control, light grey = 50 µM ANAVEX2-73, dark grey = 100 µM ANAVEX2-73.

Taken together, the in vitro and in vivo data so far clearly show that the Sig-1R agonist ANAVEX2-73 induces autophagy, as indicated by autophagic flux measurements. This encouraged us to further look into the functional consequences of autophagy induction, focusing on the impact of the degradative pathway on proteostasis in vivo. Therefore, we employed human Aβ42-expressing worms characterized by a time-dependent paralysis, due to the accumulation of Aβ42 oligomers and high molecular weight aggregates in body wall muscle cells [50,51]; it is stressed here that Aβ42-expressing worms are not considered as a model for AD, but rather as an experimental model for general proteostasis stress and proteotoxicity, where protein aggregation in muscle cells leads do a clear-cut phenotype (here, paralysis) [40]. Aβ42 protein aggregates were stained in situ with thioflavine. Compared to control worms, treatment of Aβ42-worms with ANAVEX2-73 reduced the number of thioflavine-positive Aβ42 aggregates (Figure 4A), suggesting that the induction of autophagy impacts on proteostasis, presumably by an enhanced clearance of Aβ42 aggregates, resulting in a reduced tissue deposition of aggregates. The accumulation of Aβ42 aggregates in the muscle cells is known to lead to an enhanced paralysis of the worms over time [40,50]. To analyze the impact of ANAVEX2-73-induced autophagy on the time-dependent movement behavior, the extent of this paralysis was investigated. *C. elegans* were treated with the compound (or M9 buffer as control) up to 12 days and paralysis was quantified daily. Employing two concentrations of ANAVEX2-73 (50 and 100 µM), we found a clear reduction in paralysis in the two ANAVEX2-73 treatment groups; these groups clearly separate from the controls with respect to the extent of paralysis (Figure 4B). ANAVEX2-73 clearly decelerates the paralysis rate and counteracts the time-dependent movement impairment in Aβ42-expressing worms.

Our findings that autophagy induction via a Sig-1R agonist directly impacts on proteostasis, by reducing protein aggregation and proteotoxicity-induced movement impairment in worms, suggests a possible role of Sig-1R activation in the prevention (and treatment) of neurodegeneration associated with an imbalanced protein homeostasis. Consistently with the here observed ANAVEX2-73-induced increase in proteostasis capacity, the involvement of Sig-1R deficiency or dysfunction has been described in ALS, a disorder with a highly disturbed protein homeostasis and characteristic intracellular protein aggregation. For instance, it has been shown that (1) Sig-1R missense mutation can cause ALS [24], (2) the knock-out of Sig-1R accelerates disease in superoxide dismutase 1 SOD1-mutant mice [52], and (3) an ALS-linked mutant Sig-1R causes accumulation of autophagic material and reduced autophagy [34]. Furthermore, in support of a protective role of Sig-1R activity, it was previously described that (1) treatment with the experimental drug PRE-084 improved SOD1 mice pathology [53], (2) mutant Sig-1R expression induces cytosolic ALS-linked TDP43 and FUS accumulation in cells [34], and (3) PRE-084 improves motor function and motor neuron survival in ALS mice [53]. Fully consistent with our findings, more recently, it was shown that the overexpression of Sig-1R receptor increased the number of SQSTM1/p62 and LC3B puncta, indicative of autophagy activation in human disease tissue [54].

Several steps of the autophagic processes are amenable to therapeutic modulation and different autophagy-activating compounds have already been studied at various experimental levels (in vitro and in vivo) and models of human diseases, including cancer and neurodegeneration [55]. Regarding an effective intervention of neurodegenerative disorders, of course, for any compound planned to be studied in humans in the context of the central nervous system, besides toxicity and safety issues, also the permeability of the blood–brain barrier has to be secured. One example of a compound targeting autophagy is lithium, which is in use for the treatment of bipolar disorders and is also an activator of autophagy, by interfering with upstream steps in autophagy induction. Metformin and simvastatin have also been shown, experimentally, to promote autophagy, both supposedly via the activation of AMPK, and are used for the treatment of diabetes and obesity, respectively [55]. Sig-1R agonists are under intense investigation for the treatment of different neurodegenerative diseases, including AD and ALS [15,56,57]. It is actually the combination of receptor activities that may make ANAVEX2-73 an interesting compound for AD therapy [58] and, indeed, this compound is currently in active clinical studies in both neurodevelopmental (Rett syndrome) as well as neurodegenerative diseases (AD, PD). Based on the data presented here, the use of available Sig-1R agonists to stabilize protein homeostasis by promoting autophagy may represent an added value for such a treatment approach and strongly supports further clinical studies for prevention and treatment of neurodegeneration.

Taken together, to the best of our knowledge, this is the first report that Sig-1R activation (a) enhances the autophagic flux in human cells and in *C. elegans*, and (b) has positive effects on proteostasis. We described a novel activity of the compound ANAVEX2-73 having dual selective Sig-1R/muscarinic activities in neurons. The new activity of this drug comprises a potent induction of autophagy, in vitro and in vivo, leading to an increased proteostasis capacity, and even to beneficial effects on the time-dependent paralysis phenotype in Aβ42-expressisng *C. elegans*. A specific induction of the autophagy process and a subsequent stabilization of the proteostasis in neurons represents one important concept towards the stabilization of neuronal survival and function, and may help to prevent age-associated neurodegeneration. Finally, the new finding presenting Sig-1R as a novel autophagy modulator may fuel future studies on Sig-1R, including the search for natural ligands to solve the sigma enigma.
